# The Role of Teachers in Fostering Resilience After a Disaster in Indonesia

**DOI:** 10.1007/s12310-024-09709-y

**Published:** 2024-09-05

**Authors:** Elinor Parrott, Martha Lomeli-Rodriguez, Rochelle Burgess, Alfi Rahman, Yulia Direzkia, Helene Joffe

**Affiliations:** 1 Clinical, Educational and Health Psychology, University College London (UCL), London, UK; 2 Institute for Global Health, University College London (UCL), London, UK; 3 Tsunami and Disaster Mitigation Research Center (TDMRC) and Research Centre for Social and Cultural Studies (PRISB), Universitas Syiah Kuala, Darussalam, Banda Aceh, Indonesia; 4 Tsunami and Disaster Mitigation Research Center (TDMRC), Universitas Syiah Kuala, Darussalam, Banda Aceh, Indonesia

**Keywords:** Resilience, Schools, Teachers, Disaster, Trauma

## Abstract

Disasters are distressing and disorientating. They often result in enduring community-wide devastation. Consequently, young people may seek support from trusted adults to scaffold their emotional responses and to support their psychosocial recovery. An important non-familial adult in a student’s life is their teacher. However, few studies have examined teachers’ perspectives on the support they provide to students after exposure to disasters, such as earthquakes and tsunamis, particularly in low- and middle-income countries (LMIC) with collectivistic cultural orientations. Given the potential for teachers to foster students’ resilience, the goal of this study was to examine how teachers conceptualise their role following a major disaster. Forty teachers were interviewed from three schools in Central Sulawesi, Indonesia, after a major earthquake and tsunami in September 2018. Thematic analysis shows that teachers act as agents of community resilience after a disaster. The two themes presented converge on support-based aspects. Teachers provided: (1) psychoeducational support (i.e. supporting students’ well-being and educational continuity, including encouraging their return to school) and (2) practical support (i.e. assisting administrative roles, aid distribution and disaster risk reduction). Within these themes, socioculturally specific practices are elucidated, including the Indonesian value of mutual assistance (‘*gotong royong*’), storytelling (‘*tutura*’) and the role of religiosity as a form of psychosocial support. Overall, our results highlight the capacity and willingness of teachers to play a central role in the psychosocial recovery of students and their families, contributing to community resilience. We identify implications such as the importance of providing accessible psychological training and support for teachers.

## Introduction

Young people are vulnerable to the negative psychological and behavioural manifestations of disaster-related trauma (Kar, 2009a, 2009b; Peek, 2008). The adverse psychological impacts of disasters for young people can range from transient psychological distress and poor mental health to chronic psychopathology (Norris et al., 2002). This includes post-traumatic stress disorder (PTSD), depression, anxiety and externalising behavioural problems, including substance abuse (Pfefferbaum et al., 2015). The mental health of young people can be impacted by the initial disaster exposure, as well as the cumulative stressors that disasters trigger (Chen et al., 2023), which can persist for many years post-disaster (Brown et al., 2017). Disaster stressors include threat to life, bereavement, loss of the home and widespread community social and economic disruption (Chen et al., 2023).

While trauma reactions can cause severe and long-term impairment for some, a large proportion of disaster-exposed youth initially experience elevated trauma symptoms, which diminish over time, without the need for clinical intervention (Bonanno et al., 2010; Hechanova & Waelde, 2017; La Greca et al., 2010). For example, research indicates that chronic symptom elevations are rarely found in more than 30% of young people (Bonanno et al., 2010). While a range of risk and protective factors influence the mental health of young people following disaster exposure (see Masten & Motti-Stefanidi, 2020; Masten, 2021), the social resources within a young person’s support network can effectively buffer some of the negative psychological impacts of a disaster (Silove, 2005). Particularly, in low- and middle-income countries (LMICs), resource constraints can limit the specialist mental health support available (Patel et al., 2013), causing young people to rely on friends, caregivers and teachers (Masten & Narayan, 2012). It is therefore essential to examine the factors within a young person’s environment that facilitate recovery after a disaster.

### The Role of Schools and Teachers in Supporting Resilience After Disaster

Schools are an important source of support for trauma-exposed young people. In disaster settings, schools support students across the disaster preparedness, response and recovery phases (Mutch, 2014). Teachers are expected to communicate disaster preparedness and risk reduction information and implement disaster drills (Ronan et al., 2015). During the response phase, which occurs immediately following the disaster in the emergency period, teachers are responsible for making potentially lifesaving decisions if the disaster strikes during school hours (Ema, 2012). Teachers often coordinate the repurposing of schools as evacuation shelters and/or centres for disaster relief (Ema, 2012; Oktari et al., 2015). During the recovery stage, when the focus is on restoring pre-crisis conditions, the ongoing social support from peers and teachers in schools can act as a protective buffer against negative disaster impacts (Bikar et al., 2021; Masten, 2021; Masten et al., 2021; Parrott et al., 2023a, 2023b). Furthermore, since 2000 school enrolment rates have increased drastically in LMICs (Fasih et al., 2018). For example, in Indonesia, junior secondary school enrolment has risen from 60% in 2000 to 78% in 2015 and senior secondary school enrolment has increased even more drastically from 39% in 2000 to 60% in 2015 (Fasih et al., 2018). Therefore, schools provide the social infrastructure to access a large number of children, making them a convenient site for post-disaster mental health interventions (Fazel et al., 2014).

While the role of the teacher may vary depending on the context, in general, initial teacher training does not include specialist mental health support (Shelemy et al., 2019; Shepherd et al., 2013). Nevertheless, following a disaster, teachers may receive training to deliver universal (i.e. not targeted based on the diagnostic screening of a specific clinical group) classroom-based interventions, which offer a cost-effective solution to address the substantial demand for mental health support (e.g. Coombe et al., 2015; Lai et al., 2016; Seyle et al., 2013).

A comprehensive account of the role of schools after the 2011 Canterbury earthquakes in New Zealand (Mutch & Gawith, 2014; Mutch, 2013, 2015a, 2015b, 2018) involved schools recording their earthquake stories in-depth between 2012 and 2014. Mutch (2015a) argues that teachers’ pivotal role in supporting students after disasters is under-recognised as teachers become “*quiet heroes*” (pg. 77), putting personal needs aside to demonstrate calmness, courage and empathy; many teachers prioritised the needs of their students before their home situations while navigating a chaotic post-disaster school environment. Building on this, Mooney et al., (2021) examined how the school context and in-school relationships supported students’ affective coping after the Canterbury disaster and found that teachers were trusted providers of student support. Similarly, in a comparative case study of school responses in the immediate aftermath of the 2011 disasters in Christchurch and Japan, O’Connor and Takahashi (2014) found that “*communities of care*” (pg. 52) were fostered in schools, as teachers prioritised the recovery needs of students, students’ families and the school, which strengthened school–community relations.

Together, these studies outline the critical role that schools and teachers play in supporting the psychosocial recovery of students after they have experienced collective trauma. This role may be particularly central in LMICs due to a scarcity of accessible professional mental health support (Saxena et al., 2006; Seyle et al., 2013). Despite this, little attention has been given to how teachers conceptualise their role and whether they consider providing psychological support for students to be within their domain of responsibility, particularly during longer-term disaster recovery (e.g. during the years post-disaster). Few studies have been conducted in LMICs, despite the greater burden of mental health needs and disaster impacts occurring in these settings (Kar, 2009a, 2009b).

Furthermore, much of the existing research has taken place in cultures with norms of individualism (e.g. New Zealand) rather than collectivism (e.g. Indonesia); individualism characterises societies where ties between individuals are loose, whereas ties in collectivistic societies are tight (Hofstede et al., 2010). Notwithstanding other cultural differences that may exist between such societies, collectivistic societies tend to value cooperation, social equity and acting to meet the needs of their in-group (Hofstede et al., 2010; Steel et al., 2018). These values may influence how teachers conceptualise their role in supporting students during and after a disaster. Therefore, while elements of the findings from New Zealand may apply to other highly seismic regions, including Indonesia, there are likely to be elements of teachers’ roles that are culturally specific (‘emic’) and cross-culturally applicable (‘etic’) (Triandis, 2002). O’Connor and Takahashi (2014) provide valuable insights into the care-based role of the schools in Japan, a country characterised by a collectivistic cultural orientation. However, it is crucial to recognise that these findings may not generalise to LMICs. The focus of this paper is on how teachers in Indonesia, a LMIC with norms of collectivism, view their roles and responsibilities following a devastating earthquake, tsunami and landslide.

### Resilience After Disaster

Aspects of the theory of resilience are used in this paper to guide an understanding of the role teachers play in the post-disaster recovery of their students’ and the wider school community. According to the multisystem resilience framework, an individual’s capacity to function positively and adaptively following adversity (i.e. to demonstrate resilience) is the product of reciprocal interactions between overlapping systems (Masten et al., 2021; Ungar & Theron, 2020). These range in scale from the micro-level of an individual’s biological and neurological processes to macro-level social, cultural and political systems (Masten, 2019). In post-disaster contexts, this comprehensive lens is useful for understanding the multifaceted and heterogenous resources that can influence an individual’s resilience (see Lomeli-Rodriguez et al., 2024; Masten, 2021).

From this perspective, a crucial wider system that influences the resilience of young people is their community. Although definitions often vary, ‘community’ is most commonly conceptualised as the grouping of individuals and the social structures within a geographically defined region (Rasanen et al., 2020). However, communities can be dynamic and not exclusively tied to geographical boundaries (Burgess & Mathias, 2017). For instance, disaster survivors may also belong to faith-based and/or diagnostic communities (Burgess & Mathias, 2017; Parrott et al., 2023a, 2023b). This study focuses primarily on the school community, encompassing the school environment and associated groups, including teachers, students and their caregivers.

Thus, alongside the multisystem perspective, we consider resilience at the level of the community. Community resilience is most often defined as an ‘adaptive capacity’ that supports the community to cope with adversity (Berkes & Ross, 2013; Norris et al., 2008). An influential model (Norris et al., 2008) theorises successful community resilience as a process emerging from four adaptive capacities: social capital (e.g. received and perceived social support and sense of community), economic development (e.g. equity of resource distribution), information and communication (e.g. trusted sources of information) and community competence (e.g. community efficacy and empowerment). The theory recognises community strengths and their ability to manage the demands associated with the stressor of a disaster (Gil-Rivas & Kilmer, 2016). Nevertheless, it has been argued that community and individual resilience are interlinked, particularly in disaster settings, as the availability of community resources (e.g. continuity of care providers, security of attachments) can predict an individual community member’s ability to ‘bounce back’ more than intraindividual level traits (Ungar, 2011).

In past research, scant attention has been paid to resilience at the community level compared to the vast psychological literature on individual level resilience (Berkes & Ross, 2013). Nevertheless, community resilience is increasingly garnering academic and policy attention (Patel, 2022). This lens addresses some of the criticisms of focusing on individual resilience, including neglecting contextual factors and adopting a Western, neoliberal approach that may be unsuitable for use in LMICs (Schwarz, 2018). Furthermore, examining resilience at the level of the community is valuable in disaster settings, due to the community-wide impacts of disasters and the collective responses mass disasters provoke (see Parrott et al., 2023a, 2023b). Therefore, it offers an important lens for understanding how community strengths can support young people to cope with adversity in LMICs and beyond.

### The Present Study

To address the gaps identified in the literature, this study sets out to understand the lived experiences and perspectives of teachers regarding their role in supporting students after a major disaster that occurred in Central Sulawesi, Indonesia.

Conducting research in Indonesia offers an opportunity to explore the country's intricate sociocultural and educational landscape. Given Indonesia's strong collectivistic culture, tight-knit societal connections and highly religious orientation (majority Islam in Central Sulawesi), the findings are likely to reveal nuanced insights specific to the region, facilitating the generation of contextually relevant knowledge and providing an understanding of how teachers navigate these contexts to provide effective support.

This focus is particularly pertinent due to the region’s geographical vulnerability to seismic events, which increases inhabitants’ likelihood of exposure to multiple natural hazards throughout their lifetime (Seyle et al., 2013). Specifically, Central Sulawesi is prone to a range of disasters triggered by earthquakes, tsunamis, landslides, flash floods and wildfires (Astuti et al., 2021). By gaining a deep understanding of the lived experiences of educators in this disaster-prone region, this study can bolster resilience efforts by informing the design of interventions tailored to the context. The findings may pertain to similar contexts too: collectivistic, highly religious, hazard prone LMICs.

Beyond teachers’ academic role, enshrined in Indonesian Law (20/2003), teachers are implored to act as role models to promote students’ positive *character* to shape them into good citizens based on five core values: integrity, religiosity, nationality, independence, and mutual cooperation (Nurhayati, 2020). Furthermore, teachers in Indonesia play a pivotal function in fostering close communication and collaboration with students’ families and the broader community (Oktari et al., 2018). Following a disaster, schools in Indonesia, like those in other LMICs, are likely to be supported by NGOs (Empatika, 2018). These specific dynamics of Indonesian school practices may influence how teachers conceptualise their role after a mass disaster.

The focus of this study is a major disaster that occurred in Central Sulawesi on September 28th, 2018. The disaster involved an initial earthquake that triggered tsunami and liquefaction. It was devastating: 4340 died, over 211,000 were displaced from their homes and 1,299 schools were affected (Pemerintah Provinsi Sulawesi Tengah, 2019). The widespread impact of the cascading disaster makes it an important context in which to study the role of teachers after a disaster.

While our previous work has examined the perspective of students regarding the role of their school and teachers after the same disaster (see Parrott et al., 2023b), this study focuses on the teachers’ perspectives of their own role. To our knowledge, no previous study has used interview data from disaster-affected teachers to qualitatively examine their post-disaster role in a LMIC with a strongly collectivistic culture. In doing so, we aim to elucidate the impact of the teacher’s role on students’ resilience after a disaster. Through this approach, we seek to explore teachers’ *subjective* experiences of their role, including the aspects most salient to them, rather than focusing on the factual accuracies of their post-disaster narratives.

This will be explored through the research question: How do teachers conceptualise their role following an earthquake/tsunami/liquefaction disaster?

## Method

### Participants

Three school sites in the Palu region of Central Sulawesi were selected to participate in the research. All three are public junior secondary schools (known as *Sekolah Menengah Pertama* or *SMP*), typically accommodating students aged 13 to 16. Variability in the ages of enrolled students between individual schools can be attributed to delayed entry into formal schooling due to familial responsibilities and economic activities. These schools are secular institutions, operating under the authority of the Ministry of National Education, as opposed to Islamic schools, which are supervised by the Ministry of Religious Affairs in Indonesia.

Each school was impacted by a different element of the disaster. School A is situated in a rural area in the Sigi District, which is prone to liquefaction, located relatively close to the liquefied area of Petobo. The school experienced structural damage, resulting in around 50% of the buildings either collapsing or being severely damaged. Concerns regarding the school’s structural integrity are ongoing. School B is located in the urban area of the capital city, Palu, approximately 2 km from the coastline of Palu Bay. The area was impacted by the earthquake, resulting in moderate damage, including the fence collapsing, as well as cracking and partial collapse of buildings. However, the buildings have remained functional. School C is located in an area prone to tsunamis, situated on the coastline of Palu Bay. Although the single-story buildings were not structurally damaged, the tsunami led to a loss of items including desks and chairs and large-scale mud deposition. Over time, sea water has caused corrosion of the buildings. As the school is situated in the region considered to be disaster-vulnerable (the ‘red zone’), it is at risk of relocation.

From these three sites, 40 disaster-affected teachers were selected at random by the school principal, contacted by their school and invited to participate. Demographic details related to the sample are shown in Table 1.

**Table 1:** Participant demographics

Sample characteristics		
Age	Years	27–59 (*M* = *49, SD* = *8.70*)
Gender	Female	87.5%
	Male	12.5%
Religion	Muslim	95%
	Christian	5%
Highest education completed	Postgraduate degree	80%
	None of the above	18%
School Site	Site A	42.5% (n = 17)
	Site B	30% (n = 12)
	Site C	27.5% (n = 11)

### Procedure

Interviews were conducted between January–April 2022 in the teacher’s school with a local research assistant (RA) present. The RAs provided an iPad to conduct the interviews online via Zoom with three Indonesian researchers. This approach offered numerous benefits, as it eliminated the need for the researchers to travel, which enabled data collection to be flexible according to the teachers’ schedules. The presence of a RA in person mitigated some of the disadvantages of online interviews, such as a lack of emotional support post-interview, consent challenges and technological elitism (Keen et al., 2023). All interviews were video and audio recorded to ensure a comprehensive documentation of the interview process, including capturing both verbal and non-verbal cues, resulting in accurate transcripts. Each interviewer received training on the interview technique and feedback on at least one pilot interview from the Principal Investigator (PI), who devised the now widely-used method (Joffe & Elsey, 2014). Prior to the interview, participants read an information sheet outlining the nature of the study and a consent form was signed. Lastly, participants completed demographic data and a series of psychometric scales (reported elsewhere). This study was approved by the University College London research ethics committee (Project ID 0525/001).

### Data collection

**Fig. 1:**
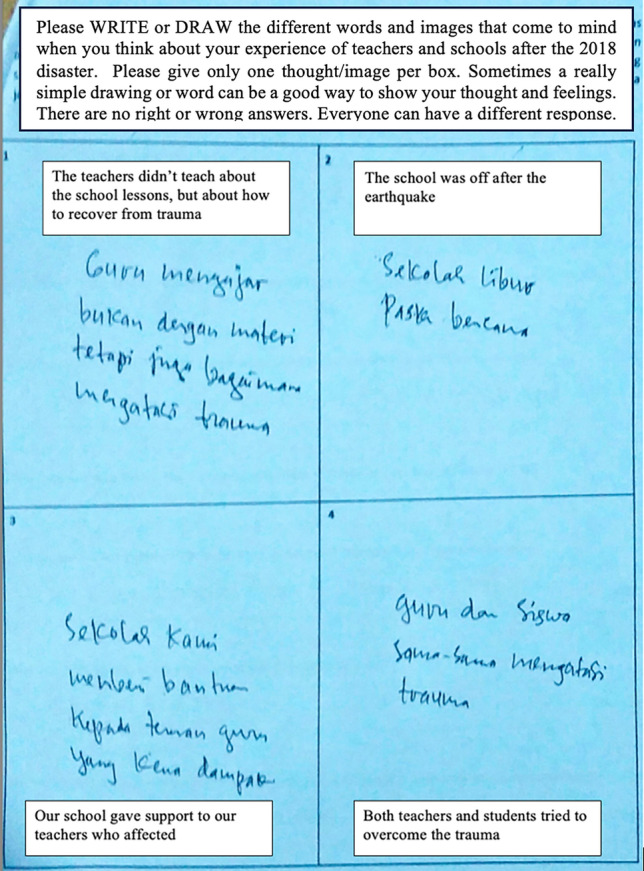
A completed grid

A free association task termed the ‘Grid Elaboration Method’ (GEM) was used (see Joffe & Elsey, 2014) Participants were given an A4 sheet of paper featuring a grid of four boxes, above which a prompt stated: ‘*Please write or draw your first thoughts and feelings regarding the role of teachers and schools in responding to the 2018 earthquake*.’ Participants were asked to give one idea or image per box and told that there was no right or wrong answer. Figure 1 shows a completed grid (with the English translations in the text boxes). Materials were translated into Bahasa Indonesian and interviews were conducted by Indonesian researchers. The prompt was checked for clarity by local researchers and piloted with four teachers from the same schools. The grid formed a naturalistic guide for the interview as the interviewer asked the participants to elaborate on the contents of each box in turn. Thus, the technique aims to reduce researcher interference to elicit ecologically valid material (Joffe & Elsey, 2014).

### Data Analysis

Interview audio files were transcribed verbatim and translated into English. Manual transcription was used to ensure quality and to allow for careful consideration of nuances related to the local context and dialect. The translations were reviewed by the bilingual Indonesian researchers who conducted the interviews to ensure that the translation captured the essence of the responses. Backtranslations of two interviews were reviewed to ensure consistency and fidelity to participants’ perspectives.

Thematic analysis, a method for analysing meaning-based patterns in a qualitative data set (Braun & Clarke, 2006), was conducted on the data. The specific approach to thematic analysis undertaken is in keeping with Joffe’s (e.g. Joffe, 2012; Joffe & Yardley, 2003) systematic thematic analysis technique used extensively in over two decades of her work (e.g. Joffe, 2012; Joffe & Bettega, 2003; Yun et al., 2023). To begin, the first and second author read the entire data set before inductively devising a coding frame. This approach enabled novel findings to develop naturalistically; teachers’ perspectives were foregrounded in the analysis, without reference to pre-existing theories. Themes were decided based on prevalent patterns found in the data relevant to answering the research question. Thematic networks charts were created to visually present the results of the analysis, as recommended for systematic thematic analysis (see Attride-Stirling, 2001; Joffe, 2012). The figures feature the percentage of participants who mentioned the theme and/or code, indicate the codes that constitute each theme (demonstrated by the ‘is a property of’ label on the figures) and illustrate the interconnections between the codes. All coding was conducted using the qualitative data analysis software Atlas ti. Although coding was conducted by the British authors, the Indonesian authors who conducted the interviews reviewed the themes.

Transparency in the systematic thematic analysis was achieved by creating a clearly defined coding frame, conducting a reliability test and reporting the prevalence of themes (Joffe, 2012). The first and second author performed the reliability test of the coding frame by independently double coding 10% of interviews: the inter-rater agreement was 71%, classified as substantial agreement (O’Connor & Joffe, 2020). This process promoted reflexivity as discrepancies regarding the multiple interpretations of codes were discussed by the researchers; based on this dialogue the coding frame was finalised before the entire data set was coded. While these steps provide transparency regarding how the results were determined, we acknowledge that all qualitative analysis necessarily involves interpretation and is influenced by the positionality of the researcher (Joffe, 2012). In consideration of the credibility of the results, during a subsequent intervention with the same teachers, teachers were presented with a summary of the research findings and informal collaborative discussions indicated that these resonated with their experiences. This opportunity for participants to engage with the results was influenced by ‘member checking’ procedures (e.g. see Birt et al., 2016; Kornbluh, 2015; Lincoln & Guba, 1985).

## Results

**Fig. 2:**
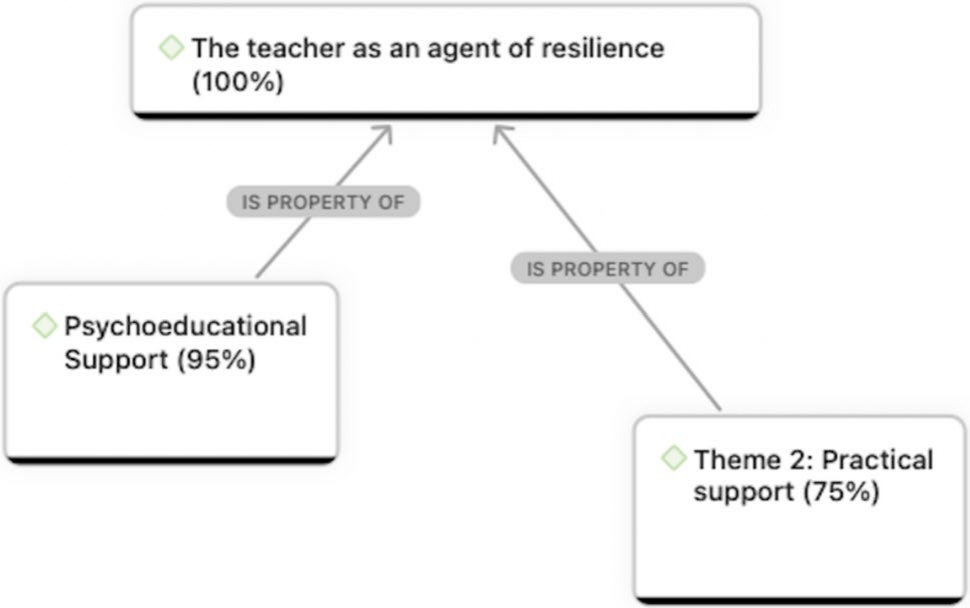
Network chart of themes

The thematic analysis led to the development of two themes that converged in terms of conceptualising the teacher’s role as an agent of resilience after the disaster. Both included a support-based aspect. According to the two themes, teachers supported students’ resilience by providing (1) psychoeducational support and (2) practical support (See Fig. 2). In elucidating each theme, its dimensions are discussed according to which were most prevalent in their interviews. Generally, teachers’ elaborations were similar across the three schools, but where differences are observed, they are highlighted.

### Theme 1. Teachers as a Source of Psychoeducational Support

**Fig. 3:**
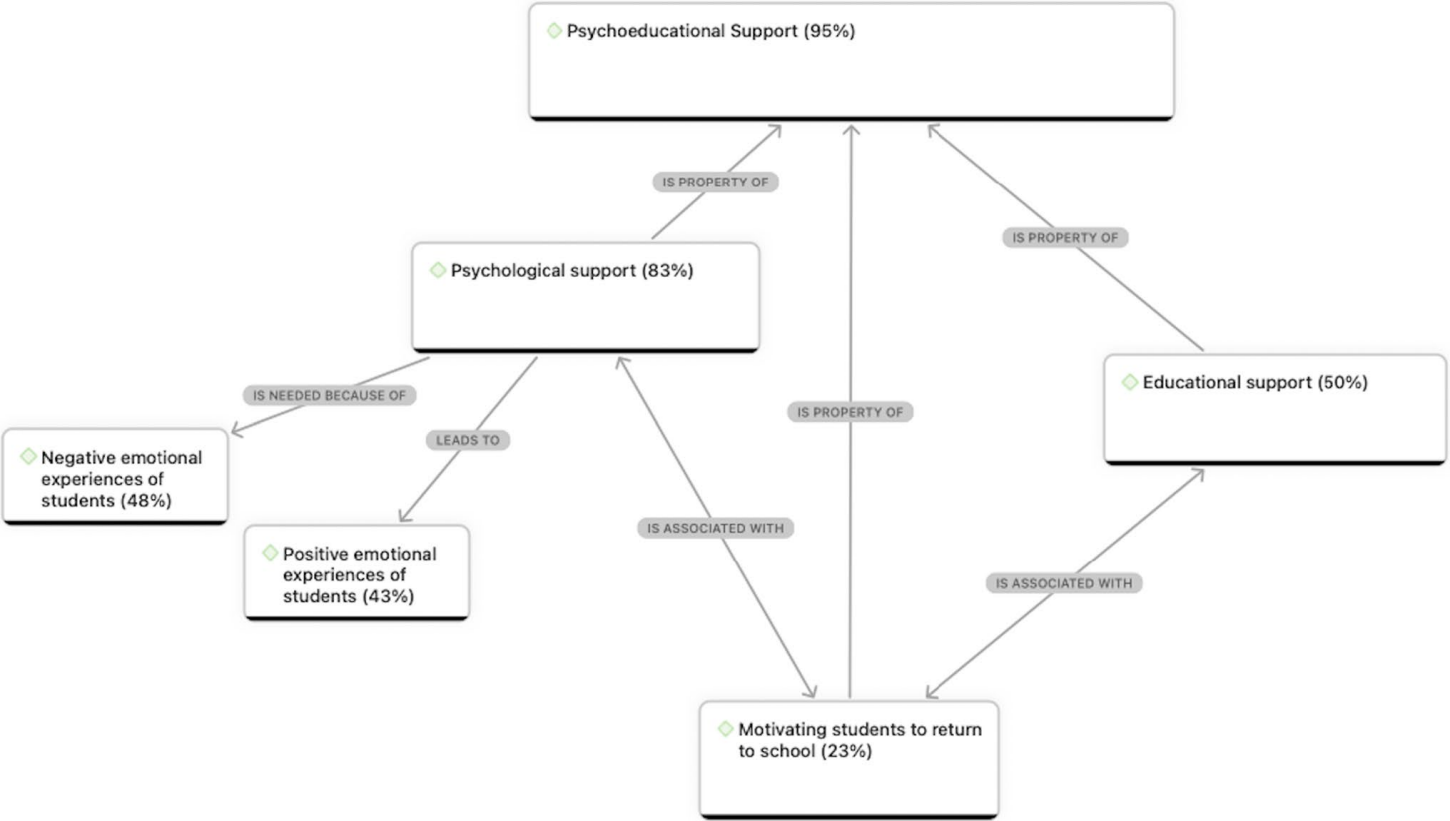
Network chart of Theme 1

The most prevalent form of teacher support after the disaster was psychoeducational (see Fig. 3). Teachers most often mentioned their role in providing psychological support to students, including one-to-one emotional support, such as listening to students’ concerns and supporting students’ emotional regulation during distress, as well as engaging students in classroom-based recovery activities. Some teachers also expressed an obligation to provide students with educational continuity despite the emergency context**.** Bridging these facets of the theme, teachers reported encouraging students to return to school to access both psychological support and academic support, to bolster their post-disaster recovery.

Many teachers supported their students’ recovery by deviating from curriculum content to provide emotional support, as they chose to suspend their usual academic role in the aftermath of the disaster. This response was driven by their concerns regarding students’ negative emotions and the adverse impact of the disaster on students’ well-being. Often the psychosocial activities offered to provide this support were arts-based, including singing, drawing, dancing and games. For example, a teacher recalled encouraging students to sing as they felt this fostered a positive classroom atmosphere and increased students’ positive affect. Emphasising this, Teacher 19 (School B, male) stated: “*Might not focus on the subject matter but focus on building an atmosphere so that the children could calm down, have fun, and slowly forget about the past they experienced*.”

Some teachers felt these activities specifically supported students who had experienced “*trauma*”. For example, Teacher 18 (School B, female) stated: *“So the ways to overcome trauma were usually by drawing, being asked to dance, sing, then playing games. Made the children even more excited to live.*”

As teachers felt responsible for supporting potentially traumatised students, some expressed a desire for further psychoeducational training, such as in “*trauma healing*”, to enhance their supportive capacity. While many teachers conveyed an appreciation for the psychological support provided by non-governmental organisations (NGOs), some teachers felt that training teachers could reduce their dependence on external support. Teacher 34 (School C, male) strongly conveyed this message: *“Schools must have teachers who are skilled in trauma healing, psychologically and can implement the disaster preparedness curriculum … If an earthquake happens in the future, they can do trauma healing activities to relieve their trauma from the earthquake and tsunami. So we don’t need to look for experts, we just need to train the teachers …”*

Furthermore, unlike NGO employees or volunteers, most teachers resided locally, in the same disaster-affected regions as their students. This facilitated the exchange of reciprocal “*stories*” (locally termed “*tutura*”), between teachers and students regarding their disaster experiences, which teachers felt was beneficial for students’ adaptive coping. This occurred both in the context of one-to-one conversations and as a classroom activity. For example, Teacher 7 (School A, female) described: *“So we look for spacious area, we studied there, we sat down… not studying but sitting, telling stories, sharing. Something like that.”*

Furthermore, teachers modelled coping strategies they believed to be beneficial, with the intention of setting an “*example*” for their students and to scaffold students’ emotional reactions. This included demonstrating emotional and behavioural regulation, as well as positive attitudes, such as being “*strong*” and “*enthusiastic*”. Some teachers felt this cultivated a positive atmosphere at school, mitigating some of the distress caused by the post-disaster situation and consequently encouraging students’ return to school. Illustrating this, Teacher 39 (School C, female) described how she aimed: *“to set an example for students that we must remain strong and enthusiastic for school.*”

Teachers also explicitly conveyed messages to students regarding the importance of their own intrapersonal psychological resources, such as “*hope*” and “*survival*”. For example, one teacher compared their own survival to those who lost their lives in the disaster, with the aim of promoting students’ hope and gratitude. Teacher 7 (School A, female) expressed: *“We are still here even [though] some of our families died, we cannot lose hope. We have to survive no matter what*…”.

Relatedly, some teachers conveyed to their students that the disaster was an opportunity for personal growth, which they encouraged by modelling and promoting adherence to desired moral standards, including religious diligence. For example, Teacher 12 (School A, female) described how the disaster provided an opportunity for students’ post-traumatic growth by “*doing good*”: “*When we taught the students, we conveyed that, in the world, we could disappear in an instant. So let's do good, don't commit immorality, don't sin, something like that happens to us suddenly. We came back alone with no one to help. So we have to do good deeds*.”

For many teachers, emotional support was intertwined with encouraging religiosity, as they felt that promoting religious messages, beliefs and practices supported students’ well-being. Occasionally, the messages endorsed the belief that the disaster was the inevitable result of “*God’s will*”, which they considered to generate “*hope*” that everything “*happened for a reason*”. Collective prayer was considered comparable to other group-based psychosocial activities, such as singing and play, as teachers felt that religious activities supported students to manage their trauma. Teacher 7 (School A, female) explained: “*So, in a tent, we were together with the students to do praying together and then play afterwards. We asked the students to sing along to heal their trauma. We asked them to play, and we joined them as well.”*

Despite teachers’ desire to model adaptive coping to their students, some acknowledged that while comforting their students, at times, they struggled to regulate their own emotions. This often stemmed from teachers’ reluctance and anxiety about being separated from their own families following the disaster. Crucially, teachers prioritised students’ needs above their own and aimed to conceal their own negative affect, such as fear and worry. Teacher 37 (School B, female) explained: “*So I rather eliminate their [students] fears and worries even though we felt scared too. It is the same for teachers and students, both experience fear."*

Teachers’ prioritisation of their students’ needs was underpinned by a sense of duty. For some teachers, this included a commitment to students’ academic development despite the challenging emergency context. This was conveyed in their enthusiasm to “*keep the spirit of teaching*”, as they were “… *still eager to teach*” in the face of disaster challenges and personal losses. Some teachers felt passionately that students’ quality of education should not be compromised due to the disaster; they believed it was their central “*obligation*” as teachers to facilitate learning. Teacher 39 (School C, female) articulated: “*It means that even though we were hit by a disaster, it does not mean that we as teachers provide a minimum education … Whatever happens, we as teachers as servants of the state still want to have the spirit to keep our students educated.”*

However, some teachers acknowledged that to best facilitate learning, they would need to adapt their pedagogy to meet the needs of the potentially traumatised students. For example, Teacher 1 (School A, female) expressed her belief that “*learning must be done in any condition*”. However, she also stressed that teachers altered their methods and expectations to be sensitive to the emotional needs of their students: “*There were many who were traumatized. Some even suffered minor injuries to their bodies. There were those whose ears were deaf at that time. So, we… I thought about how we as teachers… what methods should we use so that this child can learn and be able to get rid of the trauma immediately.”*

Due to teachers’ belief in the importance of ensuring educational continuity and the trauma-protective factors embedded in the school environment, many teachers discussed their role in supporting students to return to school. This was often mentioned alongside other ways of supporting students psychosocially, such as by facilitating positive emotional experiences to motivate the student to return to school. For example, a teacher said they “… *visited their houses, encouraging them* …”. To motivate a return to school, teachers reminded students of the collective emotional impact of the disaster. For example, Teacher 13 (School A, female) recalled telling a student: *“I said, never mind, dear, not only us who feel it but many people too. No need to mourn too much fate, no need to be sad for too long, come on, let's go to school, like that*.”

A small number of teachers expressed that some students’ caregivers were reluctant to send their children to school due to feeling anxious about family separation. However, teachers believed that these students would benefit from engaging in the psychosocial activities provided by the school, including the psychological support (“*trauma healing*”) provided by NGOs and that educational continuity was an important way to resume a sense of normality for both their students and the wider community. Some teachers felt that it was often the students who had experienced the most traumatic incidents and severe disaster losses who did not attend school. As a result, teachers targeted supporting the students they felt might be most at risk of poor post-disaster mental health by visiting their homes or displacement camps to motivate and persuade them to return to school. This is articulated by Teacher 26 (School B, female): “*Yes, only for students who were severely traumatized were visited in their houses. Given understandings, explanations to muster up their courage to go to school.”*

The imperative for students to attend school to treat their trauma before resuming learning featured as the dominant narrative in teachers’ responses. While teachers valued education, supporting students’ emotional well-being was considered a pre-requisite for students re-engaging in academic content. Illustrating this, Teacher 14 (School A, female) stated: “*We tried to dispel their panic so they could generally think again*.” Teachers communicated with parents to persuade them of the psychosocial benefits of their children returning to school. Teacher 21 (School B, female) expressed: “*We cooperated with parents and students here, there was no coercion, but we always gave motivation hoping that wherever we were, education is very important, send your children to school, no matter where you lived, if that was the case, it would stay like that, we facilitated communication for parents so that they want to get trauma healing. That was the first reason; we treated our children so they didn’t get traumatized.”*

Overall, this theme captures that most teachers conceptualised providing post-disaster psychoeducational support to students as central to their role. Teachers deviated from the curriculum to provide resilience-building activities, including sharing stories and arts-based activities, such as singing. Some teachers also encouraged religious practices, as they were considered well-being enhancing. Despite grappling with their own post-disaster mental health and personal losses, teachers prioritised students’ needs. They aimed to act as positive role models by regulating their own emotions and behaviours and demonstrating adaptive coping strategies. This enabled teachers to motivate students to return to school, to fulfil what they considered their ‘*duty*’ to teach despite the emergency context, while remaining sensitive to students’ psychological needs.

### Theme 2. Teachers as A Source of Practical Support

**Fig. 4:**
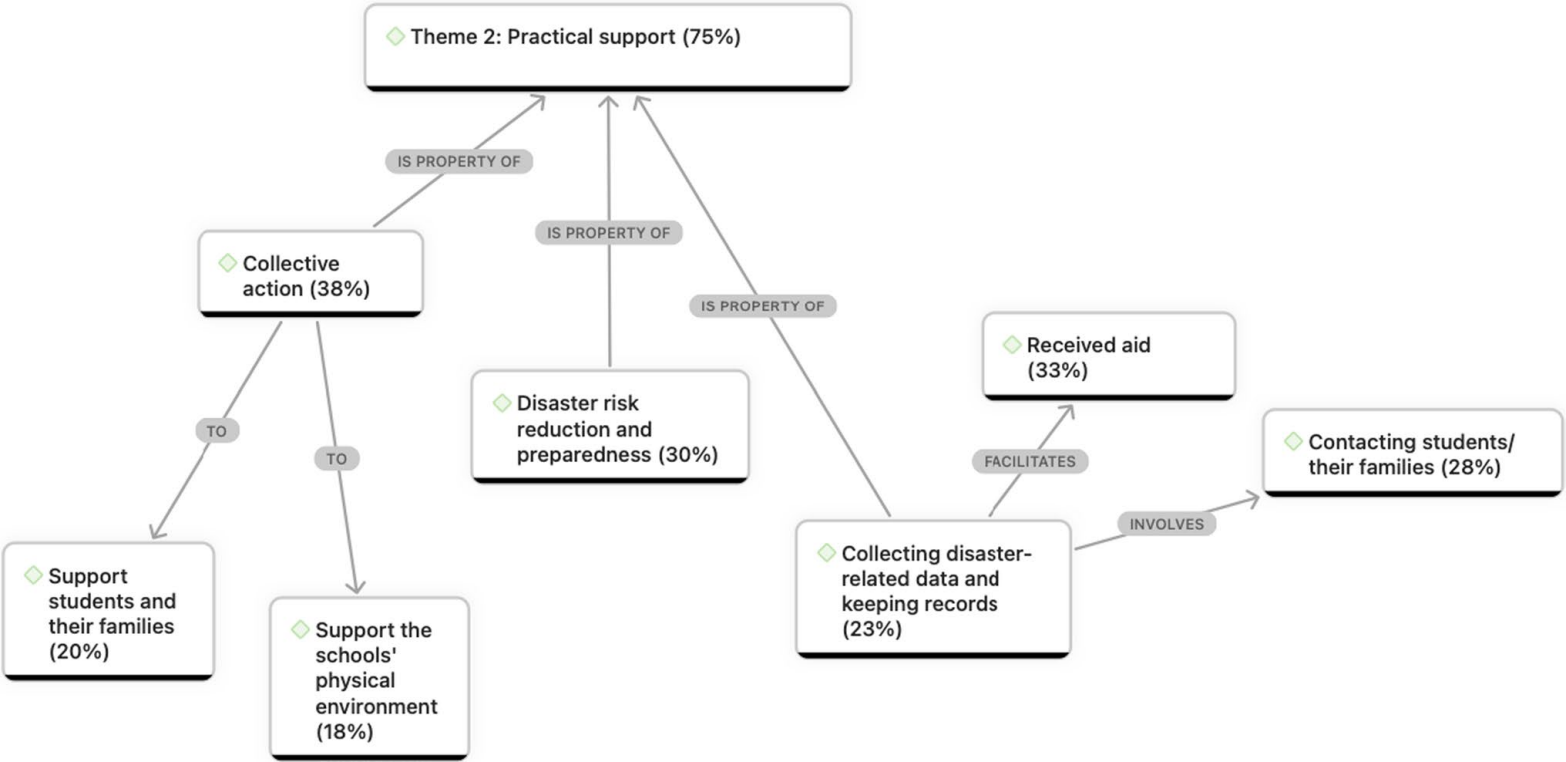
Network chart of Theme 2

A large majority of teachers described their involvement in various practical disaster recovery tasks that aimed to foster students’ resilience. The thematic network shown in Fig. 4 highlights that these tasks were comprised of collecting data and keeping records, which often involved contacting students and their families to facilitate aid distribution, mobilising collective action and promoting knowledge and action related to disaster risk reduction and preparedness.

A salient facet of the teachers’ practical role involved conducting administrative duties, such as collecting data on the disaster’s impact on students and their families. This was particularly prevalent at School A (38%) and infrequently mentioned at School B (8%). Initially, teachers attempted to contact parents by phone, but if they did not answer, they visited their homes or displacement camps. The data included keeping records of residential damage, injuries and loss of life: *“We have started to record the data at that time. So we could know how many students died, how many houses of students got washed away, lost, collapsed and slightly damaged at that time.”* (Teacher 4, School A, female). This role may have been emotionally demanding for teachers as they discovered and recorded the names of students who had died.

Teachers expressed that administrative duties were an obligation necessitated by government officials. This occurred in the immediate post-disaster response phase, as they were required to “*move quickly*” so that the government could effectively distribute aid to those in need. Although teachers fulfilled this duty, some implied that they felt a lack of agency: *“We were actually forced to attend the school because we had to list the damaged items … looked the condition of the classroom building, then list our students, anyone who lives in the affected area and then find out if they were safe or not. That's what we did initially. We finished it within a week or two after the earthquake. (Teacher 8, School A, female).”*

Despite feeling “*forced*” to gather disaster data, some teachers explained how collecting data inspired them to mobilise collective action, as it was through collecting data that teachers became aware of the school community’s hardships and disaster losses. Several teachers considered themselves responsible for mobilising collective action to assist the most severely impacted students and their families. This encompassed rallying support from other staff members and students who had experienced fewer disaster-related losses. Teacher 7 (School, A, female) reflected on mobilising support from less severely impacted students: *“So, asked those who still have some clothes, what can we donate to their friends, let’s give them something whatever it is. So, they won’t feel alone just like that. Whatever they bring me, I would share it. So, they also feel cared for.”*

Many teachers voluntarily went beyond their in-school professional requirements by visiting students in their homes and displacement camps to offer further support, distribute resources and fundraise for financial assistance. Rather than individual teachers taking responsibility for gathering support, the actions were described as a collective activity, as teachers “*came together*” to support the community and distributed donations “*from the teachers*”. At times the teachers collected the donations themselves, while at other times they organised and coordinated students to gather instrumental assistance: *“Yes, ****we****, the teachers, came together to come to them … So all this time, if there is grief, our students collect funds which we then deliver to their homes” (Teacher 14, School A, female).*

Providing instrumental support was largely motivated by a desire to reduce the emotional distress of students and their families. In response to learning about hardships that had affected the school community, such as bereavement, teachers provided tangible donations. For instance, when a teacher learnt of deaths in the community, their reaction was: “*we collected clothes, household appliance”* and another teacher took “*clothes and rice*” to bereaved parents. This aid was perceived to offer a practical advantage because of the loss of possessions during the disaster, but also symbolised community care, enabling recipients experiencing adversity to *“feel cared for*” and “*so they won’t feel alone*”. Instrumental support was also considered as an aspect of supporting students with trauma: “*Because they really really ended up being traumatized, so we shared and listened to their stories, giving clothes and other needs.” (Teacher 20, School B, female).*

Teachers at School C and School A participated in collective action to restore the physical school environment. This was especially prevalent at School C (mentioned by 45% of teachers at School C and 15% at School A), which experienced widespread deposition of sediment and other tsunami debris. Teachers and students engaged in “*cleaning up places where there was a lot of ruins*” (Teacher 15, School A, female). Some teachers expressed that cleaning was motivated by a desire to return to their original school buildings due to the uncomfortable conditions in the temporary tents. Teacher 30 (School C, female) explained that teachers collectively engaged in cleaning: “… *because it was impossible for us to continue in the tent, we slowly entered the school and cleaned it up*.”

Teachers also took responsibility for students’ safety by communicating practical disaster risk reduction advice and promoting action-oriented preparedness after the disaster, particularly during the frequent aftershocks that occurred when schools reopened. Teachers also reminded their students that the area was hazard-prone, due to the “*very active fault, Palu Koro fault*” so they should “*stay aware*”, “*alert*” and “*vigilant*” to ongoing hazards. Some teachers taught students practical advice regarding what to do in the event of a disaster, including lying under tables during an earthquake. This is illustrated by Teacher 18 (School B, female): “… *if there was an earthquake in class, our first step to save ourselves is to lie down under the table, later when the earthquake stops and then try to run and even then we have to be calm and be careful not to have a collision with other students. I gave these general instructions to students*.”

Teachers reported that they incorporated disaster-related information into the post-disaster curriculum, as they were given agency to deviate from standard curriculum content to cover material related to the recent disaster, including promoting practical action on what to do during the disaster. Teacher 14 (School A, female) exemplified this sense of flexibility in response to the circumstances: *“The material is not from the curriculum but material about earthquakes, adjusting to the situation*.”

Despite schools granting teachers agency to incorporate disaster-related material into the curriculum, few teachers discussed receiving relevant training. When training did occur, it was mentioned that this was irregular. For example, one teacher recounted attending training three years ago and while they felt this was useful, they did not retain enough information to effectively inform their students. There was also a sense of frustration at the current lack of implementation of practical disaster drills and disaster risk reduction advice in the curriculum, which would need to be mandated by the school’s senior leadership team, as teachers lacked the authority to implement whole-school activities. Teacher 34 (School C, male) expressed that he had: “*Often communicated with the principal but no realisation yet. No curriculum for disaster, I was just expressing my aspirations because I love disaster safe school activities. I like the materials, they are very interesting, and I’m very enthusiastic to participate in such activities … Maybe the school, I don’t know, due to school policies, maybe lack of coordination, I don’t fully understand, I am just a subordinate, I don’t know. I only communicated, because principal is the policy maker and the board above, because as teachers, I just do my work based on my job description.”*

As the disaster occurred outside of school hours, most teachers were not responsible for student safety *during* the disaster and most practical tasks took place during the disaster’s aftermath. However, occasional references were made to practical assistance to support student safety during the peri-disaster phase, such as when teachers encountered students during the disaster. For example, Teacher 14 (School A, female) recounted the support given to a disorientated student who ran to the school during the disaster. *“Well, this student went to the school when there was an earthquake. That’s where he felt panicked. He immediately ran aimlessly. If we see him like that, we immediately hug him. Because he survived the liquefaction of Jono Oge, he had drifted off. Someone rescued him and took him to the health center.”*

As School B was hosting a trip, some students were separated from their parents and under the school’s care. The following morning, highly concerned parents arrived at the school in search of their children. Teachers were required to navigate difficult conversations with distressed parents and help source relevant information to locate the missing students, who they later discovered had safely evacuated to higher ground. Teacher 17 (School B, female) recalled responding to parents who questioned the school’s responsibility for dealing with safety who “*came angrily and cried looking for his child*”: *“One day after the incident, I went to check the school and help panicked parents find their child who had not been home all night by contacting the principal … At that time, we just said that we were in the same situation, having a disaster. We tried our best.”*

In summary, this theme captured that a large majority of teachers considered their role to involve engaging in practical action to support students and their families, mainly during the immediate aftermath (e.g. in the days–weeks post-disaster). While some of these actions were government-mandated, teachers also went beyond the tasks school leaders had assigned them. Through mobilising collective action, teachers supported the most severely impacted by providing resources depleted by the disaster, for the dual purpose of their practical benefit and to symbolise community care. Some teachers also played a role in the physical recovery of the built school environment by clearing disaster debris, particularly at School C. Teachers also offered action-oriented disaster risk reduction advice to students to safeguard their physical needs during ongoing aftershocks and due to the hazard-prone nature of the region.

## Discussion

This study sought to contribute to a nascent area of research by gaining insight into teachers’ subjective sense of their role after a major disaster. For this purpose, 40 teachers with lived experience of a devastating disaster were interviewed using a novel free association technique. The study presented here is one of the first to hear directly from teachers in a LMIC with a culture of collectivism. In the context of ongoing seismic vulnerability and a protracted recovery, particularly given school reconstruction and reopening delays due to Covid-19, entering the perspectives of teachers three years after the disaster’s initial impact allowed for examination of the unfolding dynamics of recovery in the post-disaster years. This is important as there are temporal changes in the type of resources needed to navigate a disaster’s impacts (Kwok et al., 2016). The results of this study are encouraging, as the two themes shown in the thematic analysis collectively highlight teachers’ commitment to supporting the resilient recovery of students.

Teachers conceptualised themselves as playing a key role in supporting the psychoeducational recovery of their students. While many teachers valued providing students with a good education, most prioritised students’ post-disaster emotional well-being over resuming academic learning. To support students’ psychoeducational recovery, teachers were committed to encouraging students’ school attendance, despite the challenging post-disaster conditions. Secondly, teachers felt responsible for providing practical assistance, including support with disaster risk reduction, administrative tasks and offering instrumental support to those most adversely impacted by the disaster. Teachers’ efforts in rallying and distributing aid aimed to meet families’ tangible needs while symbolising community care.

### Teachers’ Roles in Supporting Resilience

**Fig. 5:**
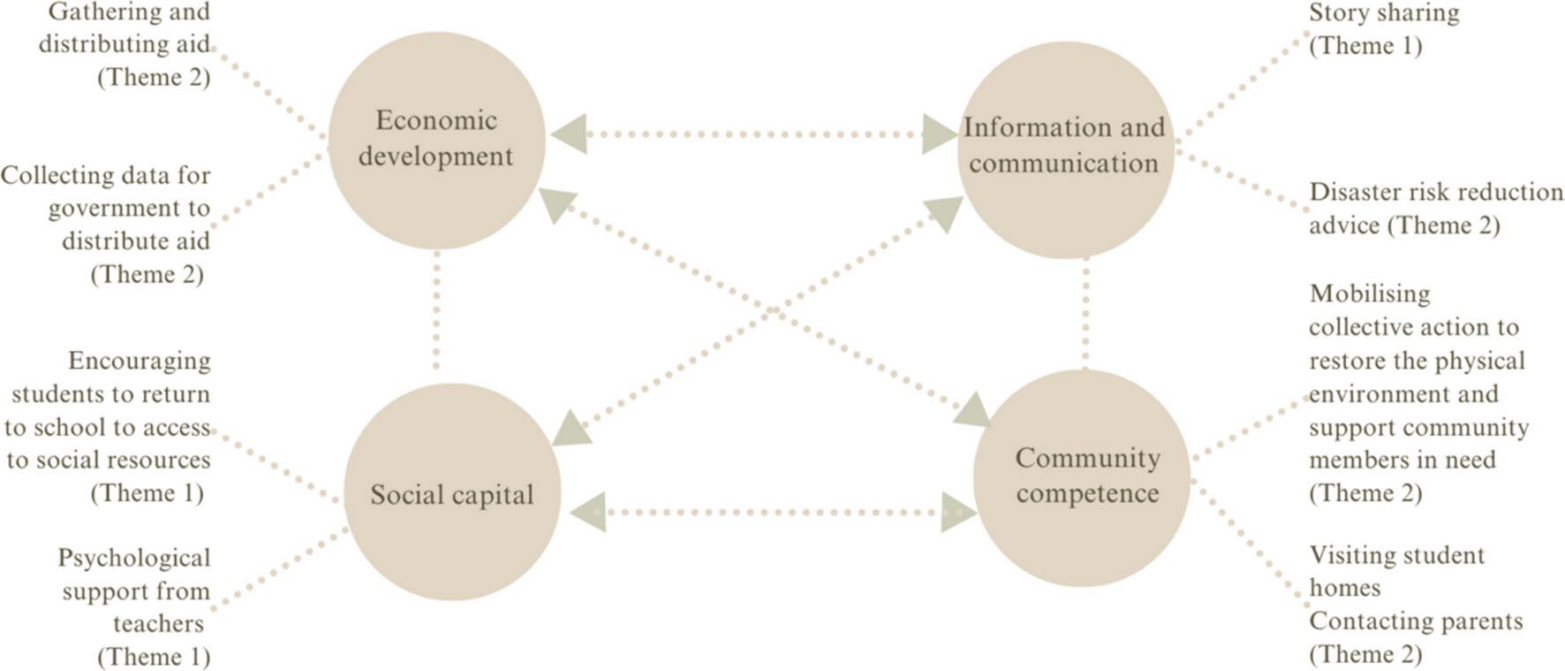
How teachers support community resilience—adapted from Norris et al. (2008)

These findings can be interpreted in relation to resilience theory. Consistent with the multisystem perspective of resilience (Masten et al., 2021; Ungar & Theron, 2020), teachers considered their role to contribute to sustaining the school system’s supportive function, potentially bolstering the resilience of individual students, such as by sharing stories and supporting students’ emotional regulation. Notably, beyond the individual level, the teachers’ most salient roles and responsibilities, through supporting students’ and their caregivers, align with the four central adaptive capacities of community resilience proposed by Norris et al., (2008). Our data indicates that teachers contributed to the resilience of the educational community through fostering: economic development, information and communication, social capital and community competence (see Fig. 5 for an adapted model). This model, and the findings in reported in this paper, are largely consistent with how the educational community (i.e. teachers, parents and students from the same school sites) conceptualised post-disaster community resilience (Parrott et al., 2023a). As individual and community resilience are often interlinked (Ungar, 2011), teachers’ roles (see Fig. 5) may benefit resilience at various levels (e.g. from the community to the individual).

Teachers’ practical interventions aided the equitable distribution of resources in the community, as teachers collected data and delivered aid, which symbolised care for their students and was felt to contribute to restoring family and child well-being through reducing post-disaster stressors (Snider et al., 2010). This instrumental support is an appropriate intervention during the initial phase of the disaster (Vernberg, 2002), to alleviate disadvantages, such as poverty, that exacerbate the mental health impacts of a disaster for the most marginalised (Hallegatte et al., 2020; Herrman, 2012). Our previous research from the same school sites found that caregivers highly valued provisions of tangible resources for building post-disaster community resilience (Parrott et al., 2023a). Additionally, teachers motivated students to return to school, which can contribute to the community’s economic recovery and a resumed sense of normalcy by facilitating caregivers to return to work (Pacheco et al., 2022). This was highly desired by caregivers in the same region (Parrott et al., 2023a).

Teachers acted as a trusted source of ‘information and communication’ by transmitting action-oriented disaster risk reduction advice and sharing stories with students about their collective experience of the disaster. Sharing stories can enhance resilience by supporting students’ emotional processing and clarifying knowledge of a confusing event (Bateman & Danby, 2013). Teachers’ willingness to listen and exchange disaster-related stories with students is connected to the cultural practice of ‘*tutura*’. Specific to the Central Sulawesi region, ‘*tutura*’ entails sharing feelings and experiences concerning challenging life circumstances, including disasters, to alleviate stress and tension. In Indonesian culture there is a prevailing norm of openness and willingness to share personal experiences with one’s in-group, as individuals seek advice and guidance from others, such as teachers, by drawing on their encounters and wisdom (Heider, 2011).

Furthermore, teachers communicating hazard awareness and preparedness can enhance students’ self and the school community’s collective efficacy by increasing confidence to deal with future disasters (Paton & Jackson, 2002). This is a crucial element of post-disaster psychosocial recovery (Hobfoll et al., 2007) and is considered by teachers, students and caregivers from the same school sites as important for building post-disaster community resilience (Parrott et al., 2023a). However, we found that teachers felt inadequately prepared and desired additional training. This is likely to be particularly pertinent to Indonesia, which despite being one of the most disaster-prone world regions, does not implement mandatory disaster education in schools (Desilia et al., 2023). To ensure that consistent and current disaster advice is enacted and transmitted by teachers, a mandatory disaster preparedness curriculum should be developed and implemented. As advice tends to be well accepted when disseminated by students to their parents (Izadkhah & Hosseini, 2005), this has the potential to further enhance the wider community’s resilience.

### A Shift in Teachers’ Roles From Academic to Social and Emotional Support

Our finding that the teacher’s role shifted from prioritising teaching academic skills towards scaffolding students’ social and emotional recovery after trauma exposure is consistent with previous research that has found an expansion of teachers’ roles and responsibilities to include supporting students with trauma (Alisic, 2012; Alisic et al., 2012). This finding also resonates with the expanding discourse of a ‘trauma informed’ approach to education (see SAMHSA, 2014), which advocates for an in-school awareness of the impacts of trauma for child development and interventions to support students who have experienced trauma, including trauma sensitive classroom practice and positive, restorative behavioural policy (Chafouleas et al., 2016; Thomas et al., 2019). Echoing this, teachers in our sample considered the impact of trauma on students’ behaviour and learning and adapted their classroom practice accordingly. This also aligns with previous research from disaster settings that finds that teachers are effective in supporting students’ psychological recovery (Mutch & Gawith, 2014), and our research from the same disaster context, which found students’ considered teachers to be a valuable source of post-disaster social and emotional support (Parrott et al., 2023b). Furthermore, the same students reflected on the importance of receiving psychological support to foster their community’s resilience (Parrott et al., 2023a).

However, we found no indication that teachers are dissatisfied with their psychoeducational support-based role, which differs from the findings of Alisic (2012) and Shelemy et al., (2019), who found that some teachers desired professionals to stick to their subject expertise (e.g. designated roles of a teacher vs a psychologist/therapist). This inconsistency may be due to the differing contexts, as the qualitative research by Alisic (2012) and Shelemy et al., (2019) took place in high-income countries with individualistic cultural orientations, where there is likely to be greater availability, accessibility and awareness of psychological professionals (Saxena et al., 2006; Seyle et al., 2013). The settings were also normative (i.e. not after a community-wide trauma), meaning that a stressor was not shared between students and teachers. In high income, mainly Western contexts, teachers tend to adopt roles based on their expertise, therefore many teachers consider students’ psychosocial well-being to be the responsibility of other professionals, such as school psychologists (Reinke et al., 2011) and social workers (Henderson Smith et al., 2023). In low-resource contexts that lack a multidisciplinary team of supportive professionals, teachers may adopt a greater responsibility for supporting students’ psychosocial well-being.

Nevertheless, when in-school professional psychological support was available, such as from NGOs, we found teachers considered this beneficial. This differs from findings in other LMICs, such as a qualitative study on survivor responses to a flood in Sri Lanka, which found a low demand for psychological services such as counsellors and psychologists, as participants preferred traditional healers (Ekanayake et al., 2013). Instead, teachers in Palu considered themselves as one component of a multi-tiered system of psychological support, which is consistent with the conceptualisation of a teacher’s role in a ‘trauma informed’ school (Chafouleas et al., 2016). Teachers mentions of *“trauma healing”* suggest that more ‘Western’ clinical psychology concepts related to trauma have been incorporated into the teachers’ local knowledge systems.

### The Influence of the Sociocultural Context on Teachers’ Roles

Our findings concerning the care and pastoral support teachers exhibited towards students and their families are consistent with some previous research from disaster contexts in HICs with individualistic orientations (e.g. Mooney et al., 2021; Mutch, 2015b; O’Connor & Takahashi, 2014) and other collectivistic post-disaster contexts, including elsewhere in Indonesia (Seyle et al., 2013) and in Malaysia (Bikar et al., 2021).

However, facets of how teachers conceptualise their role are likely to have been shaped by Indonesian cultural values and context-specific social norms, as has been observed in the way individuals cope following disasters (Ekanayake et al., 2013). In collectivistic cultures, such as Indonesia, teachers may be more willing to go beyond their professional duty, as group-oriented tasks that involve cooperating with others for a collective outcome are often well-being enhancing (Rego & Cunha, 2009; Triandis, 2000; Triandis et al., 1988). This resonates with our finding that teachers voluntarily engaged in activities that allowed for collective agency, such as rallying donations and travelling into the community to offer practical support to in-need families and to encourage students to return to school. This demonstrates values that are strong in collectivistic cultures, such as closeness and loyalty to members of one’s in-group (Hofstede, 1980, 2011; Hofstede et al., 2010) and those specific to Indonesia, including the ancient indigenous value of mutual assistance (“*gotong royong*”). This practice, reciprocally supporting one another with practical and emotional needs (Slikkerveer, 2019), is deeply rooted in the local fabric of Central Sulawesi and was highly valued by teachers, parents and students for fostering community resilience after the same disaster (Parrott et al., 2023a). These culturally embedded approaches to navigating life circumstances, including the aftermath of disasters, represent invaluable assets that could inform broader implementation strategies and dissemination efforts across Indonesia, particularly in regions grappling with similar adversities.

Despite this relational orientation, some teachers expressed dissatisfaction arising from their obligation to partake in government-mandated duties to collect data during the aftermath of the disaster. These administrative obligations, particularly at School A, located in a highly devastated region, required teachers to be away from their families in moments of fear and uncertainty. Arguably, the respect for hierarchies and social duties competed with the high value for kin-based obligations and family unit to support coping characteristic in collectivistic cultures (Schwartz et al., 2010), to create a tension that negatively impacted teachers’ sense of agency and capacity to fulfil their various social roles. This demonstrates the importance of conducting culturally sensitive research specific to disaster settings in LMICs; future cross-cultural research would benefit from elucidating whether facets of teachers’ resilience-promoting actions, such as the forms of agency and self-efficacy enacted, are unique to countries with a culture of collectivism.

Overall, we expand on the growing body of research on empowering teachers to support student mental health (e.g. see Franklin et al., 2012; Shelemy et al., 2019) by revealing that teachers in low-resource contexts may be well-prepared to undertake a support-based role post-disaster. Teachers’ willingness to support their students suggests that they hold the potential for reducing the care gap for mental health disorders, which is substantial in LMICs such as Indonesia (Setiyawati et al., 2014). Teachers may be particularly suited to this role as they are trusted, familiar adults and the school is a non-stigmatising site for mental health intervention (e.g. in comparison to mental health services).

Task-shifting, such as by training teachers to support students’ psychosocial needs, can address the shortage of mental health providers in marginalised communities (Tol et al., 2012). As teachers are from the students’ community, their support will likely align with local community values and build on existing community strengths, which Westernised, top-down solutions to improving global mental health have been criticised for ignoring (Summerfield, 2012). For example, teachers in our sample encouraged engagement in religious rituals, such as prayer to support students’ emotional recovery, comfort and sense of community cohesion. This is likely to be influenced by the highly religious nature of Indonesian society, as emphasised by the incorporation of ‘religiosity’ into the principles of character education that schools are mandated to promote (Nurhayati, 2020). Reflecting this, in our previous study, students, teachers and their caregivers from the same school sites conceptualised religiosity, including maintaining trust in God and collective devotional acts, as fostering community resilience (Parrott et al., 2023a). While these findings may be influenced by the Indonesian educational and sociocultural context, they may generalise to other highly religious contexts. As teachers’ knowledge and coping strategies have developed from a combination of professional training and personal experience, they should be empowered to provide support that is both evidence-based and resonates with students’ worldviews, local support systems and indigenous strengths.

The power of teachers to influence students’ mental health and recovery could be particularly strong within the Indonesian cultures; however, ensuring teachers receive evidence-based training that aligns with salient cultural constructs and values is essential. For instance, Indonesia scores highly on ‘power distance’ (Hofstede, 1980, 2011; Hofstede et al., 2010), meaning that authority figures are respected and often go unquestioned. Consequently, students may indisputably absorb advice from their teachers that could be detrimental to recovery. For example, some teachers promoted culturally driven narratives of fatalism: the belief that the disaster’s effects are beyond their control, being ‘Gods will’. Fatalism may support disaster-affected individuals to cope with unpredictable and uncontrollable situations (e.g. after flooding; see Ekanayake et al., 2013), but can also diminish more empowering coping strategies and reduce preparedness for future disasters (Paton et al., 2010; Solberg et al., 2010). Consequently, when teachers lack relevant training, their well-intentioned efforts may not always encourage students to engage in the most effective coping mechanisms. Therefore, teachers must be knowledgeable about appropriate, evidence-based psychosocial support and feel empowered to confidently distribute this knowledge to their community.

### The Emotional Demands of Teachers’ Roles

While it is likely to be advantageous that teachers are willing to support students experiencing traumatic stress, this finding must be underpinned by the recognition that teachers face a potential emotional “*cost to caring*” (Figley, 2013, pg. 1). In disaster settings teachers are at high risk of emotional exhaustion and burnout (O’Toole, 2017). Our finding that teachers attempted to conceal their own negative emotions to model positive coping could be beneficial for teachers’ coping, as keeping busy by engaging in work and supporting others practically has been found to provide psychological and emotional distraction post-disaster (Ekanayake et al., 2013). However, previous research also demonstrates that when teachers are required to balance personal disaster impacts with returning to work, they often experience poor emotional well-being and depression (Devaney, 2009; O’Toole & Friesen, 2016). As the stressor is shared between students and their teachers, hearing students’ disaster stories could also trigger trauma memories (Ehlers et al., 2004). Therefore, teachers would benefit from psychological support and tools to manage their own mental health needs while simultaneously supporting their students.

## Implications and Directions for Future Research

These findings have implications for school disaster preparation and response. As teachers are willing to offer psychosocial support, the development of a national training package for psychosocially informed resilient recovery enhancement would be beneficial, to provide post-disaster support for teachers while also building their capacity to effectively support their students. It is important for teachers to feel competent in supporting students’ post-disaster mental health, particularly as quantitative research has demonstrated that competence can mediate teacher’s attitudes towards providing psychosocial support (Kos et al., 2006). While numerous toolkits exist to provide teachers with relevant information regarding supporting students who have experienced trauma (e.g. Traumatic Stress Toolkit for Educators by the American National Child Traumatic Stress Network, 2008; Toolkit Child and Trauma, Alisic, 2010 in the Netherlands; Critical Incidents in Educational Communities Guidance, UK Trauma Council, 2023), these most often refer to supporting students with intrapersonal trauma, rather than following a community-wide disaster. Furthermore, these toolkits should be adapted the specific cultural context to ensure their acceptability and relevance (Gil-Rivas & Kilmer, 2016).

In addition, while toolkits exist, teachers may be unaware of these (Alisic, 2012). Therefore, future work should consider how to best tailor existing trauma-informed resources to support schools in particular contexts following disasters and increase teachers’ awareness and use of these resources. Furthermore, building the capacity of teachers through training may increase teacher’s efficacy and reduce their dependence on external supports, such as NGOs, which our results indicate teachers desired. This is consistent with the numerous recommendations in the literature to build resilience through harnessing local knowledge to empower communities to foster their own recovery (Gil-Rivas & Kilmer, 2016; Snider et al., 2010).

## Limitations

Our results must be considered in the context of some limitations. Teachers’ retrospective reporting (40–44 months post-disaster) may have led to recall bias. Furthermore, although a strength of the GEM is the minimisation of content inserted by the interviewer, we recognise that certain aspects of teachers’ roles may not have been articulated by the teachers as they are taken for granted. This may explain why fewer teachers discussed mentioning their academic responsibilities compared to psychosocial support.

Although teachers were selected at random and invited to participate, it is possible the sample is skewed as the teachers who agreed to participate may have been more interested in providing psychosocial support and therefore be more likely to go beyond academic responsibilities than those who declined to participate. Similar potential sampling bias has been found to lead to an over-reporting of trauma symptoms post-disaster, as those experiencing a greater psychological impact are more likely to be interested in participating in relevant research (Bonanno et al., 2010). In the present study, although numbers of teachers who declined participation were not recorded, the reasons provided were multifaceted, including personal commitments and scheduling conflicts. For instance, during the recruitment phase, several teachers expressed interest in participating but later faced challenges in aligning their schedules. Moreover, unforeseen circumstances, such as the recent passing of relatives, rendered some teachers unable to attend. Future research should ensure a record is kept of the teachers who decline to participate and their reasons for declining to assess potential sampling bias.

Furthermore, as women and older teachers were overrepresented in our sample, we may not have captured the male and young teacher’s experience sufficiently; future research may consider using a gender and age balanced sample to explore potentially gendered/age-based teaching roles. Additionally, this study focused solely on teachers’ perspectives. To further elucidate teachers’ roles in fostering community resilience, future research should compare the results of this study with the perspectives of other community members, such as religious leaders, students’ families and school principals.

## Conclusion

It is widely recognised that a young person’s resilience following trauma exposure depends on the functioning of dynamic systems, including their school (Masten et al., 2021; Ungar, 2011) This study provides a novel and important contribution to the understanding of teachers’ conceptualisations of the role they play in supporting students’ recovery after a major disaster in a LMIC, collectivist culture. In doing so, this work builds on existing literature that finds schools to be resilience-promoting environments, particularly for the most disadvantaged (Ungar et al., 2019). They can foster a sense of post-disaster normality and recovery (Pacheco et al., 2022; Parrott et al., 2023a, 2023b). This study emphasises the centrality of teachers for community resilience-building; they are committed, willing and resourceful and put their own needs aside to provide key emotional, social and tangible resources to promote students’ and the community’s resilience post-disaster. It is often not acknowledged that teachers go beyond their role in times of crisis (Mutch & Peung, 2021; Mutch, 2015a). Therefore, this paper advocates for a recognition of the emotionally demanding dual role of teachers in providing support for others while navigating their personal disaster stressors, affirming the need for teachers to receive post-disaster psychological support and training.

## Data Availability

The data are not publicly available due to containing sensitive information that may compromise the privacy of research participants. The data are stored privately in the University College London (UCL) repository.
